# Biological Augmentation Using Electrospun Constructs with Dual Growth Factor Release for Rotator Cuff Repair

**DOI:** 10.1021/acsabm.4c02006

**Published:** 2025-02-27

**Authors:** Yaping Ding, Yao Huang, Fucheng Zhang, Lei Wang, Wei Li, Hélder A. Santos, Luning Sun

**Affiliations:** † National Engineering Research Center for Nanomedicine, College of Life Science and Technology, Huazhong University of Science and Technology, Wuhan 430074, P. R. China; ‡ Drug Research Program, Division of Pharmaceutical Chemistry and Technology, Faculty of Pharmacy, University of Helsinki, FI-00014 Helsinki, Finland; § Department of Orthopedics, Sports Medicine Center, Affiliated Hospital of Nanjing University of Chinese Medicine, Nanjing, Jiangsu 210029, P. R. China; ∥ Department of Biomaterials and Biomedical Technology, The Personalized Medicine Research Institute (PRECISION), University Medical Center Groningen (UMCG), University of Groningen, Ant. Deusinglaan 1, 9713 AV Groningen, The Netherlands

**Keywords:** rotator cuff tear, electrospinning, bilayer structure, growth factors, tendon-bone interfaces

## Abstract

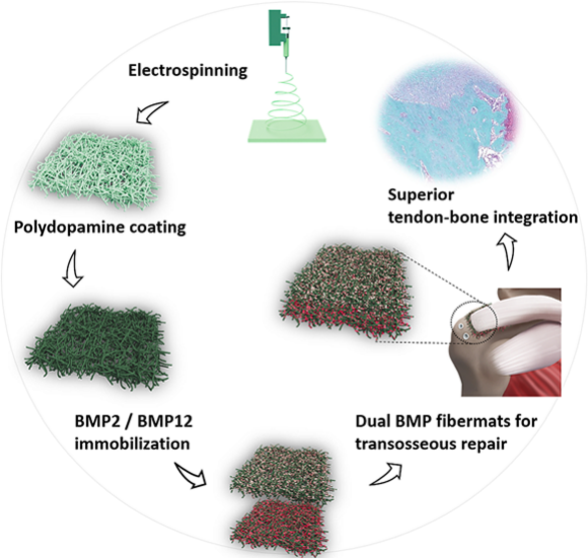

Surgical reattachment of tendon to bone is the standard therapy for rotator cuff tear (RCT), but its effectiveness is compromised by retear rates of up to 94%, primarily due to challenges in achieving successful tendon-bone enthesis regeneration under natural conditions. Biological augmentation using biomaterials has emerged as a promising approach to address this challenge. In this study, a bilayer construct incorporates polydopamine (PDA)-mediated bone morphogenetic protein 2 (BMP2) and BMP12 in separate poly(lactic-*co*-glycolic acid) (PLGA) fiber layers to promote osteoblast and tenocyte growth, respectively, and intermediate fibrocartilage formation, aiming to enhance the regenerative potential of tendon-bone interfaces. The lower layer, consisting of PLGA fibers with BMP2 immobilization through PDA adsorption, significantly accelerated osteoblast growth. Concurrently, the upper BMP12@PLGA–PDA fiber mat facilitated fibrocartilage formation and tendon tissue regeneration, evidenced by significantly elevated tenocyte viability and tenogenic differentiation markers. Therapeutic efficacy assessed through *in vivo* RCT models demonstrated that the dual-BMP construct augmentation significantly promoted the healing of tendon-bone interfaces, confirmed by biomechanical testing, cartilage immunohistochemistry analysis, and collagen I/II immunohistochemistry analysis. Overall, this combinational strategy, which combines augmentation patches with the controlled release of dual growth factors, shows great promise in improving the overall success rates of rotator cuff repairs.

## 1 Introduction

A rotator cuff tear (RCT) occurs when there is partial or complete breakdown of the tendon-bone interface in the shoulder, resulting from either rupture or degeneration. This condition leads to considerable shoulder pain and significant dysfunction in shoulder movement for patients.^1^ Surgical intervention involving direct suturing of tears is commonly performed to repair ruptures, often yielding successful clinical outcomes. However, there is a notable risk of retear, ranging from 26 to 94% depending on the size of tears, which is often attributed to incomplete reconstruction of the interface tissues at the damaged sites.^2,3^

The tendon-bone interface, known as the enthesis, comprises four continuous gradient zones: tendon, fibrocartilage, mineralized fibrocartilage and bone.^4^ After direct surgical suturing, the natural healing process typically progresses through an inflammatory phase followed by remodeling and repair phases. However, this often results in the formation of scar-like tissues and weak tendon-bone integration, leading to reduced biomechanical strength and a high retear rate.^1,5^ Consequently, besides conventional surgical interventions, various strategies such as biocompatible augmentation mats, growth factor delivery, or cell therapies have emerged as promising approaches to enhance tendon-bone integration.^3,6,7^ Studies indicate that biocompatible mats can function as a temporary interface between the bone and tendon, bridging gaps and expediting the healing process;^3^ while, growth factors have shown the ability to enhance cellular responses, including cell recruitment, proliferation, and differentiation, thus contributing to improved performance at the healed interface.^8^ Despite these advancements, various strategies come with inherent limitations. For instance, solely relying on a biocompatible film may be insufficient in stimulating cell differentiation for a closer integration of the tendon-bone interface. Similarly, the direct injection of growth factors might struggle to maintain therapeutic efficacy in the long term due to their short half-life. The healing process of RCT involves intricate dynamic interactions among osteoblast cells, tendon cells, and multiple growth factors, emphasizing the necessity for combinational strategies to enhance healing outcomes.^1^

Electrospun porous films have been extensively investigated as augmentation mats in repairing RCT.^5^ Their ultrathin fiber diameter and ultrahigh porosity enable them to closely mimic the extracellular matrix (ECM) during tissue regeneration. Additionally, the high surface area of electrospun fibers provides numerous sites for attaching growth factors. Depending on the interaction dynamics, absorbed growth factors can be released from the scaffolds either rapidly through physical absorption or in a sustained manner when covalently bonded.^9^ One effective method for binding biomolecules to electrospun fibers involves coating the fiber surface with polydopamine (PDA), garnering widespread interest in controlled drug delivery.^10^ The oxidation of catechol and amine groups enables dopamine to self-polymerize onto almost any surface, forming a PDA adhesive layer.^11^ This PDA coating serves as a binder between the fibers and molecules, facilitating a steady release of the bound molecules.^12,13^

**Figure 1:**
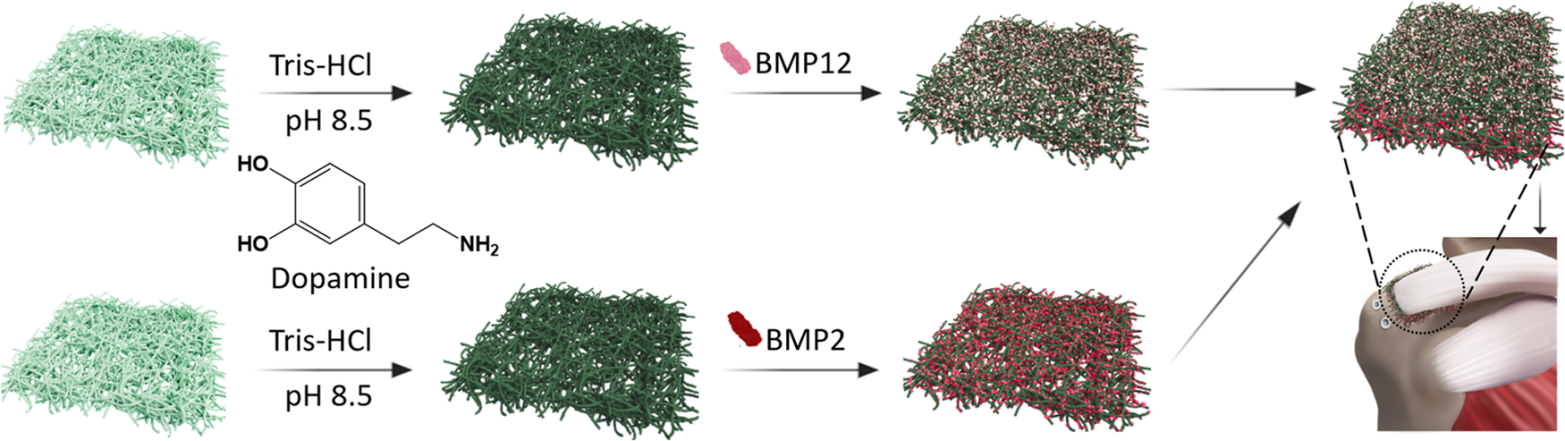
Schematic illustration of the bilayer film facilitating the delivery of dual growth factors for repairing RCT.

In this context, to fully leverage the benefits of both augmentation mats and biomolecules, we propose a combinational approach utilizing a bilayer construct of electrospun films to facilitate the controlled release of dual growth factors for repairing full-thickness RCT. This strategy aims to improve tendon-bone integration and maximize clinical outcomes. Poly(lactic-*co*-glycolic acid) (PLGA), an FDA-approved biopolymer, was selected as the polymer matrix for fabricating fibrous mats due to its well-known biocompatibility and biodegradability. Two growth factors, bone morphogenetic protein (BMP)2 and BMP12, were loaded onto the fiber surface through PDA coating for each respective layer. BMP2 was assumed to promote the growth of osteoblasts, while BMP12 was intended to stimulate the formation of tendon and fibrocartilage tissues.^14−17^ The combinational bilayer construct was strategically positioned with the BMP2 layer facing the bone tissue and the BMP12 layer facing the tendon tissue at the ruptured tendon-bone interface, followed by surgical suturing. This combinational strategy is anticipated to enhance healing efficacy, bolster the biomechanical strength of the repaired interface, and consequently reduce the retear rate, as illustrated in Figure 1.

## 2 Methods

### 2.1 Preparation of Electrospun Mats with and without BMP Immobilization

#### 2.1.1 Fabrication of Electrospun PLGA Mats and PLGA–PDA Mats

To prepare spinnable solutions, 1 g of PLGA (PURASORB PDLG 5010, Corbion, Netherlands) was first dissolved in 10 mL of 1,1,1,3,3,3-hexafluoro-2-propanol (HFIP, ≥99%, Sigma-Aldrich) overnight at room temperature. The solution was then loaded into a glass syringe for electrospinning, following previously described methods.^18^ Specifically, with a flow rate set at 2 mL/h, voltage at 15 kV, and a collecting distance of 15 cm, continuous PLGA fibers were deposited onto a metal plate. The resulting fibrous scaffolds were vacuum-dried at room temperature for 24 h to eliminate residual solvent before further treatments.

For surface modification, the as-prepared PLGA mats were immersed in a dopamine hydrochloride (Sigma-Aldrich) solution with a concentration of 2 mg/mL in 10 mM Tris–HCl buffer (pH 8.5) under continuous stirring for 24 h.^19^ Following this, the PDA-coated PLGA fibrous mats were rinsed with deionized water (DI H_2_O) to remove any excess PDA molecules.

#### 2.1.2 Immobilization of BMP2 and BMP12 onto PLGA–PDA Mats

BMP2 (Beyotime, China) or BMP12 (Beyotime, China) were initially stabilized using a bovine serum albumin (BSA, 0.1%, Sigma-Aldrich) aqueous solution. The prepared PLGA–PDA or PLGA (100 mg) mats were immersed in a solution of BMP2 or BMP12 (2 μg/mL, 10 mL) at 4 °C for 24 h, followed by rinsing with DI H_2_O to remove excess unbound proteins. The amount of BMP loaded onto the PLGA–PDA and PLGA mats was determined using enzyme-linked immunosorbent assay (ELISA) on BMP solutions before and after immersion. The resulting samples were denoted as BMP2@PLGA–PDA and BMP12@PLGA–PDA, respectively.

### 2.2 Physicochemical Characterization

The surface morphology of electrospun fibers, including PLGA, PLGA–PDA, and BMP@PLGA–PDA, was examined using a scanning electron microscope (SEM, Quanta 250 FEG, FEI). Fiber diameters were statistically analyzed using image processing software, ImageJ (NIH). Attenuated total reflectance Fourier transform infrared spectroscopy (ATR-FTIR, Nicolet 6700, Thermo Scientific) was employed to check the immobilization of the PDA coating and subsequent proteins. Each measurement consisted of 32 spectral scans within the wavenumber range of 4000–525 cm^–1^. The wettability of the prepared fibrous mats was determined by measuring the water contact angle (KSV, CAM 200, Finland), with five measurements conducted for each composition.

### 2.3 Release Studies of Immobilized Proteins

The release kinetics of immobilized proteins were investigated following methods reported in previous studies.^20,21^ Briefly, 10 mg BMP2@PLGA–PDA and BMP12@PLGA–PDA were incubated in 5 mL of PBS at 37 °C in a shaking incubator. Supernatants were collected at time intervals of 1, 4, 12, 24, 48, 72, 96, and 168 h and supplemented with fresh PBS. The concentration of the collected supernatants was quantified using ELISA according to the manufacturer’s protocol. The release studies were conducted in triplicate and cumulative release profiles were plotted against time.

### 2.4 Cellular Response of Osteoblasts to BMP2@PLGA–PDA Mats

#### 2.4.1 Cell Viability Assessment

Osteoblasts were isolated from the calvaria bones of neonatal rats using established protocols.^22^ Specifically, the procedure involved mincing the collected tissues, followed by digestion using a trypsin solution containing 0.2% collagenase and 0.1% hyaluronidase for 5 cycles of 20 min each, with intermittent shaking. Cell suspensions from cycles 3, 4, and 5 were collected, centrifuged, and resuspended in cell culture medium for further cultivation, with medium changes performed every 24 h. The osteoblasts from second passage were then cultivated in the Dulbecco’s modified Eagle’s medium (DMEM, Shanghai BasalMedia, China) supplemented with 5% fetal bovine serum (FBS, Hyclone, USA) and antibiotic solution (penicillin at 100 U/mL and streptomycin at 100 μg/mL, C0222, Beyotime, China) at a concentration of 10^4^ cells per well in 96-well plates. The cell cultures were maintained in an incubator (MCO-15AC, SANYO, Japan) with an atmosphere of 5% CO_2_ and 95% humidity at 37 °C for the indicated periods. The culture medium was refreshed every 2 days.

To assess cell viability on BMP2@PLGA–PDA mats, the Cell Counting Kit-8 (CCK-8, C0038, Beyotime, China) was employed following the manufacturer’s protocols. PLGA–PDA and PLGA were used as control groups. After culturing for 24, 48, and 72 h, cells were replenished with fresh culture medium containing 10 vol % CCK8 solutions and subsequently incubated in the dark for 1 h. Afterward, 100 μL of the mixed medium from each well was transferred to a new 96-well plate, and the optical density (OD) was measured at a wavelength of 450 nm using a microplate reader (Multiskan MK3, Thermo Scientific).

#### 2.4.2 Cell Proliferation

Cell proliferation was further observed and assessed through fluorescence staining. Osteoblasts were initially cultured in DMEM medium in a 48-well plate at a concentration of 2 × 10^4^ cells per well, with the medium refreshed every 2 days. At day 3 and day 7, the cells were washed three times with PBS, fixed with 4% paraformaldehyde (PFA, Sinopharm, China) for 15 min, and washed again three times with PBS. Next, the cell cytoskeleton was stained with phalloidin (C8001, Bioss, China), which was diluted with PBS containing 0.1% Triton X-100 at a ratio of 1:200, for 1 h in the dark. Following three more PBS washes, the cell nuclei were stained with Hoechst 33258 solution (C1017, Beyotime, China) for 5 min followed by three PBS washes. The stained cells were then observed under a fluorescence inverted microscope (IX71, Olympus, Japan).

### 2.5 Cellular Response of Tenocytes to BMP12@PLGA–PDA Mats

Tenocytes were initially isolated from rat Achilles tendon using an optimized protocol.^23^ To specifically identify tenocytes, collagen I was chosen as the marker and subjected to immunofluorescence staining. The isolated cells were first fixed in 4% PFA, underwent three washes with PBS, and were then blocked with BSA. Subsequently, the cells were incubated overnight at 4 °C with the primary anticollagen I polyclonal antibody (bs-10423R, Bioss, China), followed by three PBS washes. They were then incubated with the secondary antibody FITC-labeled goat antimouse IgG (A0568, Beyotime, China) for 1 h, counterstained with Hoechst 33258 solution (C1017, Beyotime, China), and examined under an Olympus microscope.

#### 2.5.1 Cell Viability Assessment

Tenocytes were cultured in DMEM medium supplemented with 5% FBS and antibiotic solution at a concentration of 10^4^ cells per well. The cells were cultivated under the same conditions as those described for osteoblasts above, with the culture medium refreshed every 2 days. Following a protocol analogous to that used for osteoblasts, the cell viability of tenocytes on BMP12@PLGA–PDA films was evaluated using the CCK8 kit. PLGA–PDA and PLGA films served as control groups.

#### 2.5.2 Cell Proliferation

As described above, the proliferation of tenocytes was also assessed using fluorescence staining. Phalloidin (C2201S, Beyotime, China) and Hoechst 33258 (C1017, Beyotime, China) were utilized to stain the cytoskeleton and nuclei, respectively. After being cultured on BMP12@PLGA–PDA, PLGA–PDA, and PLGA films for 3 and 7 days, the stained cells were examined under a fluorescence inverted microscope.

#### 2.5.3 Western Blot Analysis

The expression of tenogenic markers was evaluated using Western blot analysis. After culturing tenocytes on BMP12@PLGA–PDA films at a density of 2 × 10^5^ cells per well in a 6-well plate for 7 days, proteins were extracted using RIPA lysis buffer (P0013B, Beyotime, China). The extracted proteins were subsequently centrifuged at 12,000 rpm for 10 min, and the resulting supernatant was collected for total protein quantification using the bicinchoninic acid (BCA) assay kit (KGPBCA, KeyGEN BioTECH, China). Control groups included analysis of protein expression in tenocytes cultured on PLGA–PDA and PLGA films.

Subsequently, the protein extracts in each group were mixed with 5× SDS loading buffer and incubated in boiling water for 5 min. Afterward, samples were centrifuged at 12,000 rpm for 5 min, loaded onto 10% SDS-polyacrylamide gel electrophoresis (SDS-PAGE) gels, and transferred onto poly(vinylidene difluoride) membranes. The membranes were blocked with 5% skim milk in Tris-buffered saline-T (TBST) at 37 °C for 2 h, followed by overnight incubation at 4 °C with primary antibodies. After washing with TBST, the membranes were incubated with horseradish peroxidase (HRP)-labeled goat antirabbit IgG antibody (1:5000, A0208, Beyotime) at 37 °C for 1 h. Following three TBST washes, antibody detection was performed using the SuperSensitive electrochemiluminescence solution (ECL-0011, Beijing Dingguo Changsheng Biology, China) according to the manufacturer’s instructions. The membranes were then imaged using the ChemiScope5300 Pro Integrated chemiluminescence imaging system (CLiNX Science Instruments, China). All protein expression levels were normalized to GAPDH. Primary antibodies included mouse anti-GAPDH monoclonal antibody (1:20000, 60004–1-Ig, Proteintech, China), rabbit anti-SOX9 antibody (1:1000, ab26414, Abcam), rabbit anti-SCXA antibody (1:1000, DF13293, Affinity Biosciences, China), rabbit anti-TNMD polyclonal antibody (1:1000, bs-7525R, Bioss, China), rabbit anticollagen I polyclonal antibody (1:1000, bs-10423R, Bioss, China), and rabbit anticollagen II antibody (1:1000, bs-0709R, Bioss, China).

### 2.6 Animal Models for Chronic Rotator Cuff Injury and Repair

120 male Sprague–Dawley rats, aged 5 weeks and weighing 150 ± 10 g, were randomly assigned to 5 groups. The control group underwent direct repair of the detached supraspinatus tendon to its anatomic footprint (transosseous repair). In the other groups, electrospun mats were employed as augmentation during transosseous repair. All animal procedures followed a protocol approved by the Experimental Research Institute of Nanjing University of Chinese Medicine (No. ACU170706). Animals were sacrificed at 4 and 8 weeks postoperatively for analysis.

Before surgery, all rats received anesthesia *via* intraperitoneal injection of 10% chloral hydrate (0.3 mL/100 g). To induce the chronic rotator cuff injury model, the left shoulder underwent a procedure to completely detach the supraspinatus tendon from its insertion site on the humerus. The rat was positioned in the lateral decubitus position, and the shoulder joint was incised to expose the supraspinatus insertion on the humeral head. The supraspinatus insertion was then resected, with approximately 2 mm of the distal tendon of the supraspinatus excised. The shoulder skin was subsequently sutured layer by layer to complete the establishment of animal models for chronic rotator cuff injury.

At 4 weeks postsurgery, in the control group (24 rats), the animals’ shoulders underwent repair using a 4–0 VICRYL absorbable suture to reattach the supraspinatus tendon to the humeral head through the bone tunnel (transosseous repair). In the experimental group, consisting of 96 rats, transosseous repair was performed using different mats: PLGA–PDA, BMP2@PLGA–PDA, BMP12@PLGA–PDA, and a dual-BMP construct. These mats were positioned between the distal end of the tendon and the cancellous bone, with BMP2@PLGA–PDA oriented toward the bony side and BMP12@PLGA–PDA toward the tendon side.

#### 2.6.1 Histomorphometric Analysis

60 rats were sacrificed at 4- and 8-weeks postrepair surgery, respectively, and tissue specimens were collected for histomorphometric analysis. The harvested tissue specimens underwent decalcification, paraffin embedding, and subsequent coronal plane slicing to produce 5 μm sections. Prior to histological analyses, the sections were dewaxed and rehydrated. To assess the total area of new fibrocartilage formation at the insertion site postrepair, the tissue sections were initially immersed in a fast green dye solution for 5–10 min, followed by washing and subsequent immersion in a safranin dye solution for 15–30 s. The stained slices were then quickly dehydrated with alcohol, cleared with xylene, and sealed with neutral gum seal. Under light microscopy, images were captured, and the total fibrocartilage area was manually delineated by outlining the metachromasia area on the stained slides.

#### 2.6.2 Immunohistochemical (IHC) Staining of Collagen I and Collagen II

Immunohistochemical staining for collagen I and collagen II was performed to evaluate collagen formation. Initially, 5 μm sections were deparaffinized and rehydrated. Following this, sections were rinsed with PBS and blocked with 0.25% goat serum for 1 h at room temperature. Subsequently, sections were treated with a primary anticollagen I antibody (Col I, ab34710, 1:100 dilution, Abcam) and anticollagen II antibody (Col II, ab34712, 1:100 dilution, Abcam) at 4 °C overnight. The sections were further incubated with an HRP-conjugated secondary IgG antibody (1:5000, A0208, Beyotime) for 1 h at room temperature. Color reactions were developed using diaminobenzidine, followed by counterstaining with hematoxylin. The stained slides were then imaged using light microscopy.

#### 2.6.3 Biomechanical Test

To assess therapeutic outcomes, 60 animals (6 per group per time point) were sacrificed 4- and 8-weeks postsurgery for biomechanical analysis. The harvested shoulders were frozen and thawed at room temperature prior to mechanical testing. After the gross dissection, only the supraspinatus muscle and its tendon-bone insertion attached to the humeral head were retained for testing. The cross-sectional area of the repaired insertion site was measured using a digital caliper. Subsequently, specimens were securely positioned on a biomechanical tester (Instron, Boston, MA) and subjected to a 50 N load at a speed of 5 mm/min after initial preloading cycles. The strength-strain curves were recorded, and the maximum tensile load of the supraspinatus tissue was determined from these curves.

### 2.7 Statistical Analysis

The data are presented as mean ± standard deviation (SD). Statistical analysis involved a one-way analysis of variance ANOVA, using GraphPad Prism 9.3.0 software (GraphPad Software). Significance levels were set at *P < 0.05, **P < 0.01, and ***P < 0.001.

## 3 Results

### 3.1 PDA Coating Enables Sustained Release of BMPs from PLGA Fibers

Optimal augmentation patches for RCT repair are expected to not only effectively bridge interface gaps, but also feature porous architectures that enable cell infiltration and nutrient exchange, as well as bioactive cues that promote cell proliferation and maintain specific cellular activities.^24^ Attributed to the highly porous structures consisting of ultrathin fibers, electrospun fiber mats have demonstrated superiority in resembling the natural extracellular matrix, which greatly facilitate cell growth and proliferation for tendon-bone tissue integration in RCT repair.^25^

**Figure 2:**
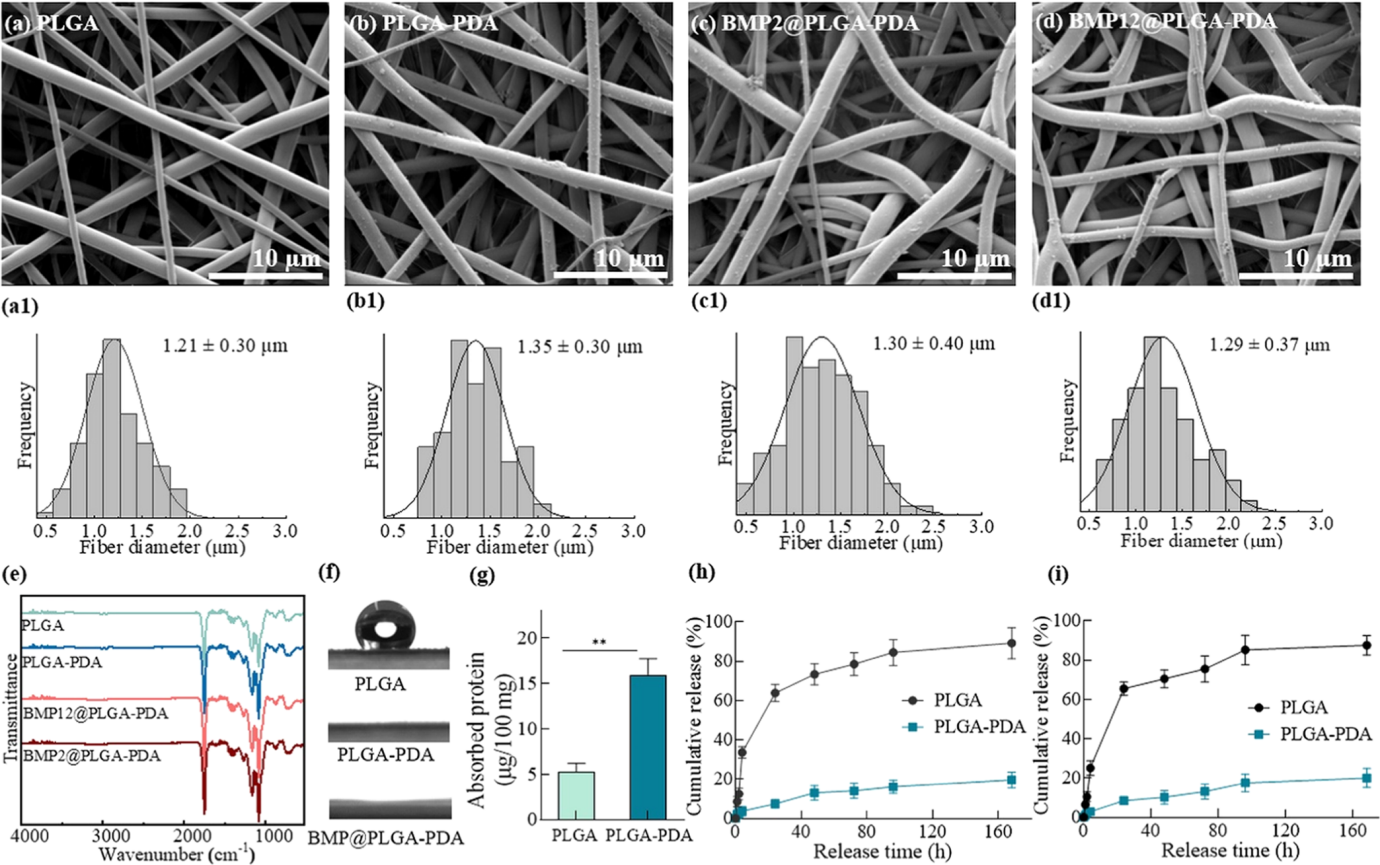
BMPs immobilization onto PLGA fiber mats *via* PDA chemistry. (a–d) Morphologies and (a1–d1) fiber diameter distributions of the as-prepared PLGA, PLGA–PDA and BMP@PLGA–PDA electrospun fibrous mats, respectively; (e) FTIR of the as-prepared electrospun fibrous mats; (f) representative images of the contact angle measurement on the fibrous mats; (g) protein absorption efficiency of pure PLGA and PLGA–PDA electrospun fibrous mats (*n* = 3,**P < 0.01); (h), (i) cumulative release profiles of BMP2 and BMP12 loaded on PLGA–PDA electrospun fibrous mats (*n* = 3).

Here, as shown in Figure 2a–d, the prepared PLGA fiber mats exhibited a typical nonwoven fibrous structure, comprising uniform fibers with an average size of 1.2 μm. The successful PDA coating, indicated by the apparent dark-gray appearance of PLGA–PDA fibers, did not significantly alter the fiber structures in terms of size and pore structure. While PLGA fibers displayed smooth fiber surfaces, the PDA-coated and BMP-immobilized fibers showed few particulate aggregations on the surface owing to the immersion process (Figure 2c,d). The thin coating layer did not result in observable differences in the FTIR spectrum of all groups (Figure 2e), suggesting that the chemical structures of the backbone fibers remained unaltered by the PDA coating or BMP immobilization. Nevertheless, the PDA coating and BMP immobilization drastically transformed the hydrophobic PLGA films into fully hydrophilic structures, as evidenced by the contact angle measurement (Figure 2f). This hydrophobic-to-hydrophilic transformation was also observed on PDA-coated PCL fibers.^26^ As reported in the literature, PDA coating could efficiently anchor biomolecules and maintain sustained release over several weeks.^27,28^ In the current study, comparable amounts of BMP2 and BMP12 were loaded onto fiber structures through PDA mediation. The absorbed proteins on the PLGA–PDA structures were nearly three times higher than those on the PLGA-only fibers and maintained sustained release profiles in an analogous manner (Figure 2g–i). In contrast, the PLGA-only fibers released more than 80% of the proteins within 72 h. The burst release within 24 h was reduced by almost 90% in the PLGA–PDA group.

### 3.2 BMP-Immobilized Fiber Mats Promote Cell Viability and Proliferation *In Vitro*

**Figure 3:**
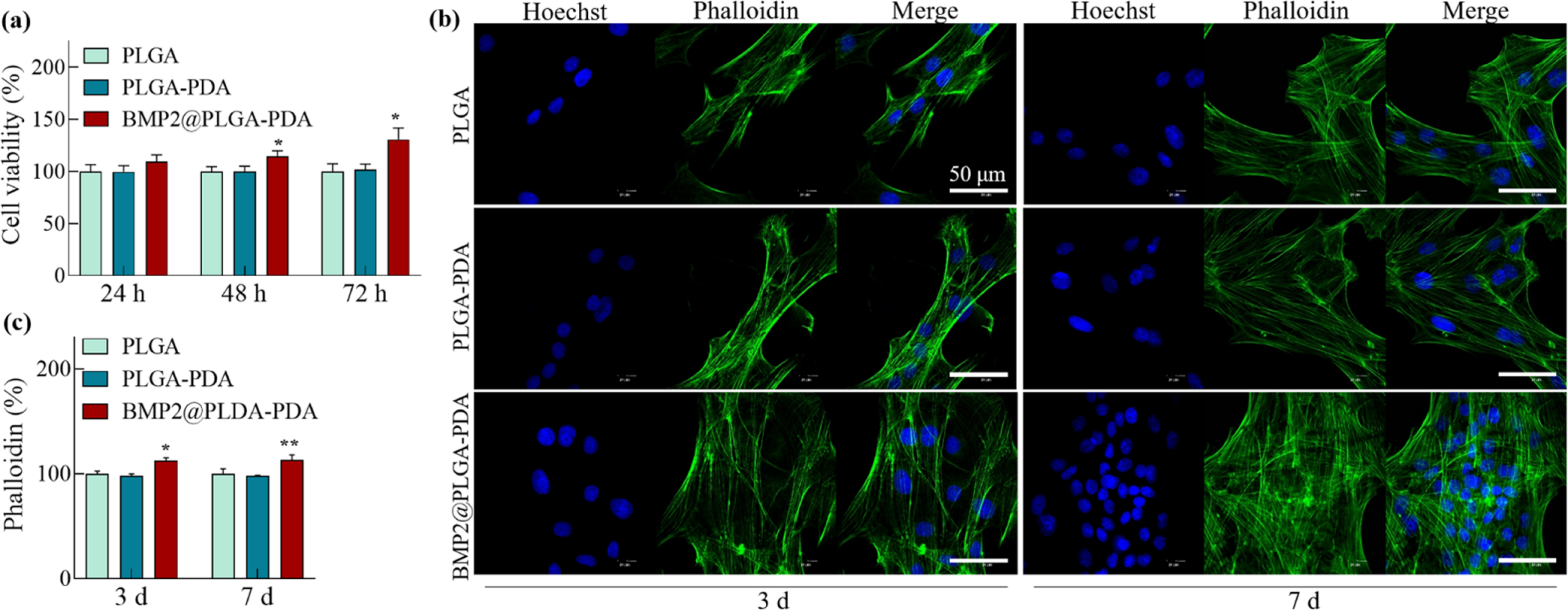
Cell viability and proliferation of osteoblasts in response to BMP2@PLGA–PDA. (a) CCK-8 analysis to qualify the cell viability of osteoblasts when cultured on fibrous mats for 24, 48, and 72 h; (b) immunofluorescence staining images of osteoblasts proliferation for 3 and 7 days on fibrous mats (green cytoskeleton was stained by phalloidin, and blue nuclei was stained by Hoechst; scale bar, 50 μm); (c) the quantitative analysis of the stained cytoskeleton. The data is presented as mean ± SD (*n* = 4, *P < 0.05 and **P < 0.01).

**Figure 4:**
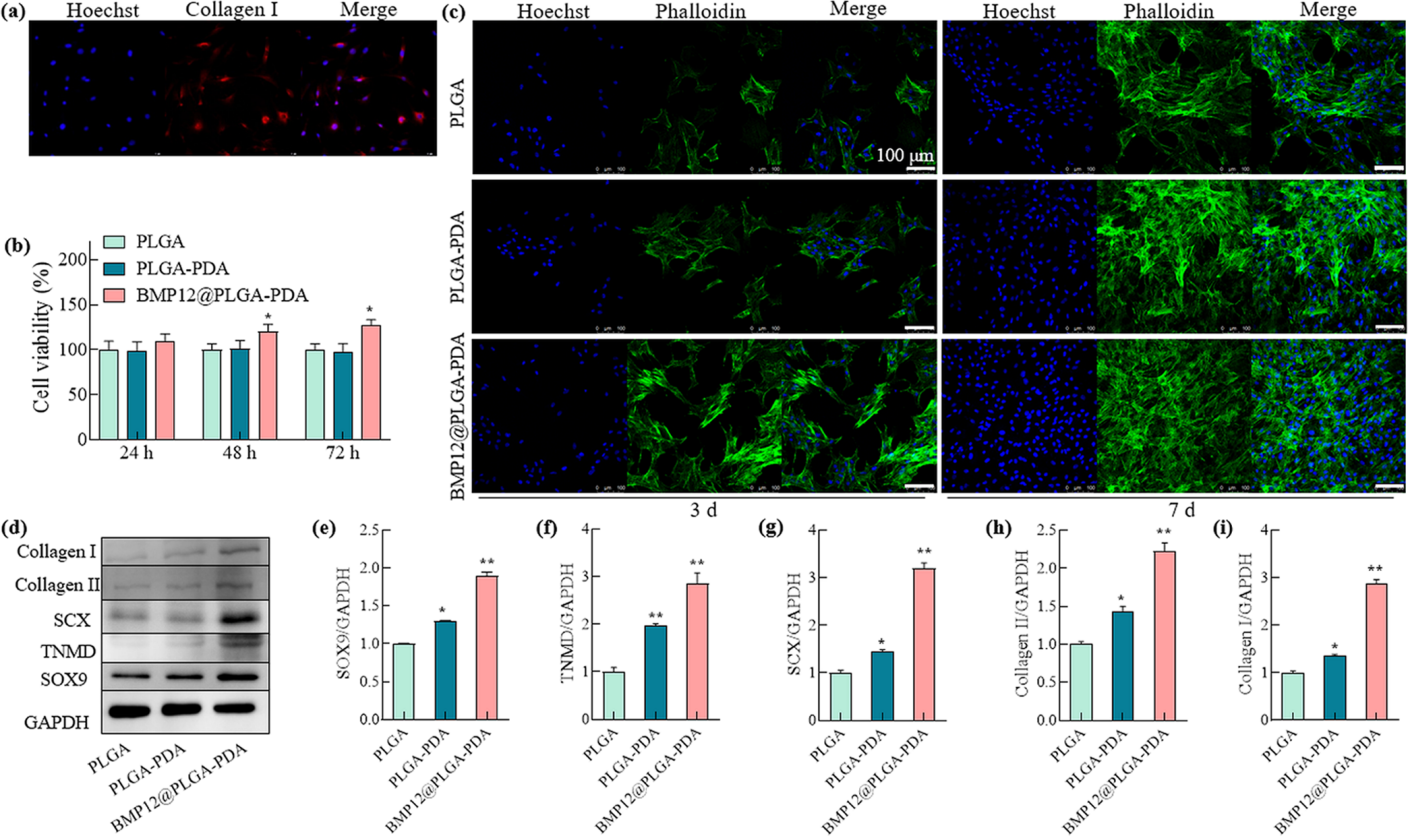
Cell viability, proliferation and tenogenic/chondrogenic markers of tenocytes in response to BMP12@PLGA–PDA. (a) Immunostaining of collagen I to identify the successful isolation of tenocytes; (b) CCK-8 analysis to quantify the cell viability of tenocytes when cultured on fibrous mats for 24, 48, and 72 h; (c) immunofluorescence staining images of tenocytes proliferation for 3 and 7 days on fibrous mats; (green cytoskeleton was stained by phalloidin, and blue nuclei was stained by Hoechst; scale bar, 100 μm); (d) western blot analysis of tenocytes cultured on fibrous mats for 7 days; (e–i) quantitative analysis of the protein expression of tenogenic/chondrogenic markers (SOX9, TNMD, SCX, Collagen II, and Collagen I) in (d). The data is presented as mean ± SD (*n* = 4, *P < 0.05, and **P < 0.01).

To promote the healing process at the tendon-bone interface, the proposed bilayer structure was designed to be inserted between the torn tendon and bone, serving as a bridging patch to expedite healing.^3^ Previous studies have shown that BMP2 efficiently promotes bone healing, while BMP12 is believed to stimulate the formation of tendon and cartilage-like tissues.^29,30^ In order to verify the potential beneficial effects of BMP2 and BMP12 on osteoblasts and tenocytes, respectively, comprehensive *in vitro* cell viability and proliferation studies were conducted, as shown in Figures 3 and 4.

As demonstrated in Figure 3a, while cell viability assessed *via* CCK-8 analysis was comparable between the PLGA and PLGA–PDA groups, osteoblasts exhibited significantly enhanced viability when cultured on BMP2@PLGA–PDA fiber mats for 48 and 72 h. Additionally, fluorescence staining of osteoblasts after culturing on BMP2@PLGA–PDA fiber mats for 3 and 7 days (Figure 3b,c), indicating a statistically significant enhancement in cell proliferation facilitated by the BMP2-immobilized fiber mat.

Regarding the tenocytes, collagen I staining was initially conducted to confirm the successful isolation (Figure 4a). The CCK-8 results (Figure 4b) showed statistically significant enhanced cell viability of tenocytes when cultured on BMP12-loaded fiber mats for 24, 48, and 72 h compared to the other two groups. Immunofluorescence staining of cell proliferation for 3 and 7 days (Figure 4c) suggested a substantial increase in tenocyte growth and proliferation when cultivated on BMP12-loaded fiber mats. Additionally, the expressions of tenogenic and chondrogenic markers, as well as collagen, in tenocytes were further assessed through Western blot analysis (Figure 4d). The markers analyzed included SRY-box transcription factor 9 (SOX9), tenomodulin (TNMD), scleraxis (SCX), collagen I, and collagen II, with glyceraldehyde 3-phosphate dehydrogenase (GAPDH) serving as the internal control. For instance, SOX9, a master transcription factor regulating multiple pathways in chondrogenesis, has been reported to guide the formation of tenocyte lineage molecular pathways and actively participate in regulating gene expression necessary for tenocyte function and the synthesis of ECM, thereby enabling the formation of strong and flexible tendons.^31−33^ TNMD plays a critical role in tenocytes proliferation and collagen fibril maturation.^34^ Quantitative analysis of the Western blot results indicated statistically significant upregulation of all the aforementioned markers in the PLGA–PDA and BMP12@PLGA–PDA groups compared to the pure PLGA group. Specifically, the expressions of SOX9, TNMD, SCX, collagen II, and collagen I in the BMP12-loaded group were approximately 1.9, 2.9, 3.2, 2.2, and 2.9 times higher (Figure 4e–i), respectively, compared to the PLGA group. The beneficial influence of BMP12 on tenogenic markers has been confirmed in other studies. For instance, Kumlin et al. reported that SCX expression, an early differentiation marker and a transcriptional activator of TNMD expression, was significantly upregulated after exposure to BMP12 (GDF7) for 1–3 days; while TNMD, a late tendon differentiation marker, increased after 3 days of exposure.^35^ Furthermore, in the study by Shukunami et al., SCX^+^/SOX9^+^ tenocytes were shown to differentiate into chondrocytes capable of establishing fibrocartilage interfaces.^31^ Consequently, due to the upregulation of tenogenic and chondrogenic markers, the expression of representative matrix proteins such as collagen I and collagen II was enhanced.

### 3.3 Dual-BMP Fiber Mats Significantly Improve Tendon-Bone Interface Integration *In Vivo*

**Figure 5:**
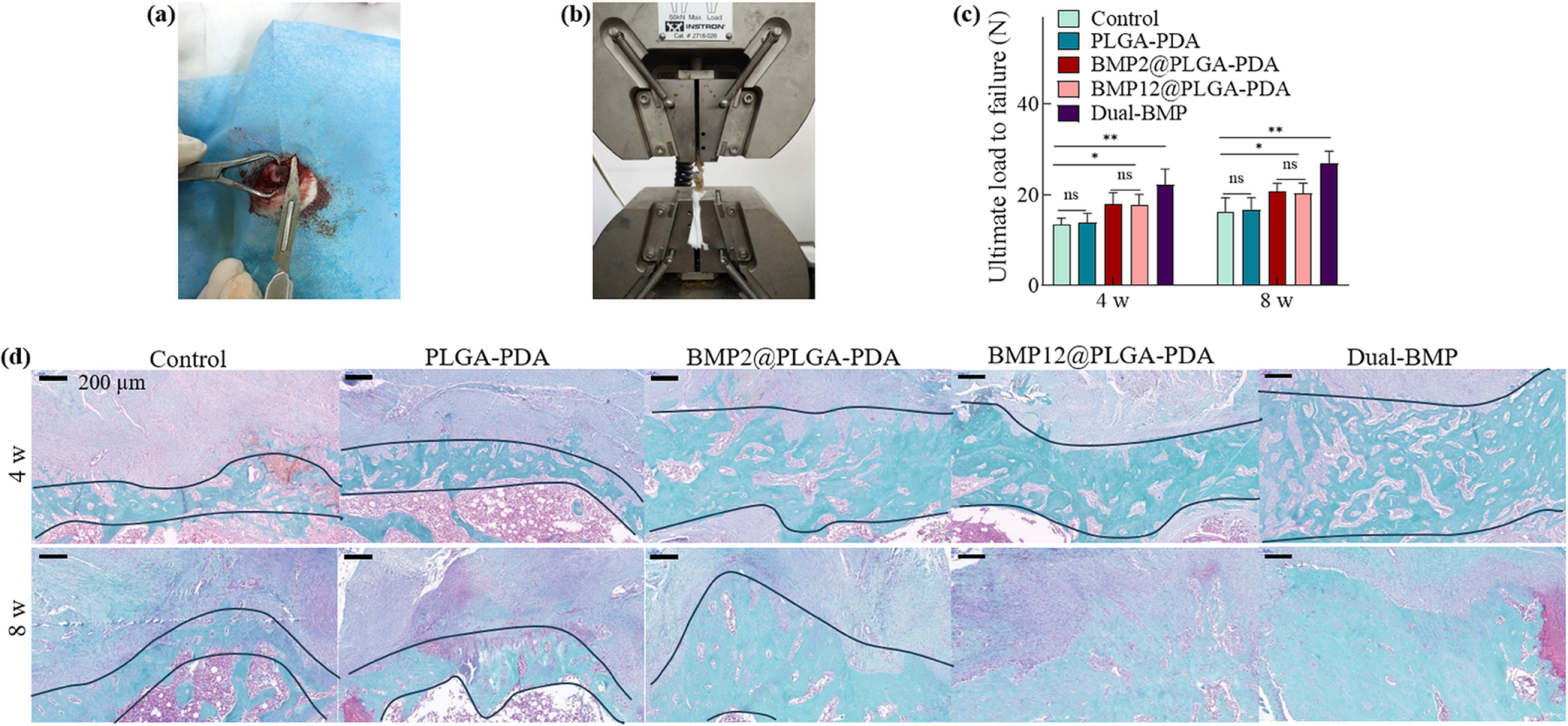
Bilayer fiber mats promote the recovery of tendon-bone interface *in vivo*. (a) Images of the *in vivo* surgery; (b) biomechanical testing of the harvested inserts and (c) comparative studies on ultimate load to failure force among groups; (d) safranin O/Fast green staining images of the recovered interface after 4- and 8-weeks post-treatment with PLGA–PDA, single BMP@PLGA–PDA and the dual-BMP mats, with direct suturing as sham control. The data is presented as mean ± SD (*n* = 6,*P < 0.05 and **P < 0.01).

To assess the potential therapeutic efficacy, bilayer fibrous mats were implanted in an *in vivo* RCT model in rats. The mats were positioned at the tendon-bone interface, with BMP2-loaded mats facing the bone and BMP12-loaded mats facing the tendon tissue, followed by suturing for augmentation (Figure 5a). The treatment efficacy was initially evaluated through biomechanical testing of the harvested inserts after 4- and 8-weeks postsurgery (Figure 5b). As shown in Figure 5c, BMP immobilization significantly enhanced the ultimate load to failure of the inserts at both time points, regardless of whether single or dual growth factors were used, compared to the control and PLGA–PDA groups. Notably, the dual-BMP group exhibited significantly superior outcomes compared to the single BMP groups. The area of newly formed fibrocartilage was delineated manually on stained images (Figure 5d). It is evident that the average thickness of the interface increased in all BMP loading groups, while the thickness in the PLGA–PDA group was comparable to that of the control group. Specifically, 4 weeks postsurgery, the interface thickness in the BMP group was more than twice that of the control and PLGA–PDA group, while it was three times higher in the dual-BMP group. The thickness and tissue density were further enhanced after 8 weeks postsurgery in the BMP group, contributing to significantly improved biomechanical performance as shown in Figure 5c.

**Figure 6:**
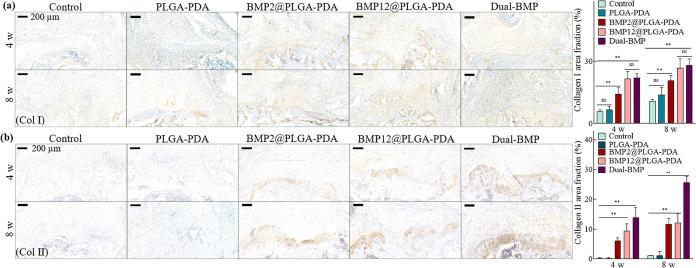
Immunohistochemical images and analysis of (a) collagen I and (b) collagen II expression after 4- and 8-weeks following treatments with PLGA–PDA, single BMP@PLGA–PDA and the dual-BMP fiber mats, with direct suturing as sham control. The data is presented as mean ± SD (*n* = 6, *P < 0.05, and **P < 0.01).

The expression of collagen I and collagen II in the healed tendon-bone interfaces was further immunoassayed at 4- and 8-weeks following treatments to investigate the influence of different treatments on the microstructure of the interface (Figure 6a,b). Specifically, collagen I, present in all four distinct zones,^4^ was significantly expressed in the BMP12 and dual-BMP groups compared to the other groups, with no significant difference observed between them. Moreover, collagen II, predominantly expressed in calcified and noncalcified fibrocartilage zones, showed a significant increase in the dual-BMP group compared to all other groups. After 8 weeks, the collagen II level in the dual-BMP group was more than twice that of the single BMP groups, indicating significantly improved fibrocartilage formation. Collagen I gradually formed in both the tendon and fibrocartilage areas, while collagen II predominantly existed in the fibrocartilage zone.

## 4 Discussion

Innovative regenerative strategies like plasma treatment, stem cell therapy and engineered augmentation patches or scaffolds have emerged to repair massive RCT tears while minimizing the risk of retearing.^7^ Among these approaches, augmentation patches are envisioned to bridge the gaps, maximize the tendon-bone contact area and provide crucial mechanical support during surgical interventions.^36,37^ However, achieving full functional restoration may necessitate additional biochemical stimulation. A combination of growth factors has shown promise in simultaneously regulating cellular pathways to regenerate bone, tendon, and fibrocartilage tissues, particularly in healing the torn tendon-bone interface.^8,38^ Various growth factors, including basic fibroblast growth factor, transforming growth factor-β, insulin-like growth factor 1(IGF-1), and members of BMP families have been delivered to promote cell proliferation and differentiation, thereby enhancing the biomechanical strength of the repaired tendon-bone interface.^8,39−41^ Nevertheless, the short half-life of these growth factors can lead to decreased bioactivity during circulation and degradation under physiological conditions when administered locally *via* intra-articular injection.^42^ Consequently, multiple injections may be required for optimal efficacy. To address these challenges, biocompatible carriers such as hydrogels, woven patches, three-dimensional scaffolds, and electrospun fiber mats have been engineered to deliver the growth factors while maintaining their bioactivity over extended periods.^8,16^ Among them, flexible electrospun fiber mats with high porosity closely mimic the ECM of native tissues, facilitating cell proliferation and growth. Moreover, these mats can sustainably release cargos, offering a promising approach for enhancing tissue regeneration and reducing the likelihood of retearing.^5,25^

Here, we propose a combinational delivery system based on electrospun fiber mats to integrate the functions of augmentation patches and prolonged delivery of growth factors. PDA chemistry, serving as a universal immobilization strategy, was utilized to load and deliver growth factors sustainably.^43^ BMP2 and BMP12 were selected as model growth factors to verify the efficacy of the proposed dual-BMP platform for restoring the tendon-bone interfaces. The release profiles indicated that with PDA chemistry, the loading amount of the growth factors has tripled, and the growth factors were sustainably released. BMP2 and BMP12 exhibited comparable release profiles regardless of the protein type, probably due to their similar molecule structures and the strong binding ability of PDA. Analogous findings were reported by Pan et al. and Gao et al. in their studies on PLGA/hydroxyapatite scaffolds for delivering BMP2 and IGF1 *via* PDA coating, and the codelivery of BMP2 and ponericin G1 by PLGA–PDA scaffolds, respectively.^44,45^ The sustained release not only extends the duration of therapy but also maintains the local concentration at the injury site, obviating the need for repeated injections. BMP2, one of the first well-characterized BMPs and an FDA-approved growth factor used in numerous clinical surgeries, has demonstrated beneficial effects on osteoblast growth, bone remodeling, and regeneration, making it extensively investigated in bone-related diseases.^46−50^ BMP12 has shown great potential for tenogenic cell differentiation by stimulating early phase tenogenic markers, such as SCX and TNMD, making it an optimal candidate for treating tendon-related issues.^8,51,52^ Thus, the combinational patches consisting of dual-BMP specifically targeted for bone and tendon tissue regeneration are expected to not only strengthen surgical suturing but also regulate the proliferation of osteoblasts and tenocytes, consequently facilitating tissue regeneration.

The cellular responses of osteoblasts and tenocytes to BMP2@PLGA–PDA and BMP12@PLGA–PDA were further assessed, respectively, using other groups as controls. Both cell types demonstrated significantly enhanced viability and proliferation when cultivated on BMP-loaded fiber mats, as evidenced by the CCK8 analysis and immunofluorescence staining results. The expression of tenogenic markers revealed by Western blot analysis further confirmed the chondrogenic potential of tenocytes when cultivated on the BMP12-loaded fiber mats. It has been reported that SOX9 activates numerous genes that affect chondrocytes proliferation and ECM deposition, particularly by directly trans-activating Col2a1, the gene expressed most strongly in proliferating chondrocytes.^53^ Therefore, the significant expression of SOX9 revealed in this study was speculated to play a crucial role in tenocytes differentiating into fibrochondrocytes,^31^ which further contribute to the formation of fibrocartilage-like ECM. As is known, the maturation of fibrocartilage is pivotal for resembling the original tendon-bone interfaces and restoring biomechanical strength.^4,5^ The significant upregulation of collagen I was also validated in the study by Lee et al.,^30^ indicating that BMP12 induces a dose-dependent increase in collagen I expression, even at concentrations as low as 1 ng/mL.

The therapeutic efficacy, commonly characterized by biomechanical testing of harvested inserts and immunostaining of fibrocartilage matrix components, such as collagen I and collagen II at the tendon-bone interfaces, was further assessed using *in vivo* rat models postoperatively. At 4- and 8-weeks postsurgery, the immunostaining analysis of the tendon-bone interface revealed significantly increased glycosaminoglycan and collagen II deposition in the dual-BMP group, indicating fibrocartilage formation rather than scar tissue. Collagen II, a major constituent of cartilage, is present in both nonmineralized and mineralized fibrocartilage zones,^54^ playing critical roles in strengthening the interfaces and reducing the retear rate of healed RCT. Consequently, the load to failure force of the dual-BMP group was statistically higher compared to other groups.

Although this strategy has shown promising outcomes in both *in vitro* and *in vivo* studies, certain aspects still require further attention. For example, beyond osteoblasts and tenocytes, various cell types, including immune cells, play crucial roles in the bone-tendon interface by regulating the microenvironment and contributing to tissue regeneration.^55^ Understanding the overall influence of biomaterials on the microenvironment and intercellular communication would provide valuable insights into the underlying regulatory mechanisms and will be a key focus of our future research.

## 5 Conclusions

Overall, this study utilized a combined therapeutic approach, integrating electrospun biomaterials with growth factors, to leverage the advantages of augmentation patches and the stimulatory effects of dual growth factors. The objective was to reinforce mechanical strength and bolster the healing potential of the tendon-bone interface post-RCT. Using PDA chemistry, the model growth factors, BMP2 and BMP12, were released in a sustained manner, significantly promoting the proliferation of osteoblasts and tenocytes, as well as the expression of chondrogenic and tenogenic markers *in vitro*. *In vivo* animal experiments revealed a notable increase in fibrocartilage formation, leading to a significant improvement in the biomechanical strength of the formed enthesis. This combined therapeutic approach, integrating both structural and biochemical stimuli, holds promise not only for rehabilitating tendon-bone interfaces but also for addressing broader challenges in interface restoration within the field of tissue regeneration.

## Data Availability

Data will be made available on request.
